# HIV and AIDS-defining opportunistic illnesses in the state of Qatar: A cohort population-based retrospective study covering 17 years (2000-2016)

**DOI:** 10.1016/j.amsu.2022.103842

**Published:** 2022-05-20

**Authors:** Maisa Ali, Almurtada Razok, Mahmoud Gassim, Nada Elmaki, Wael Goravey, Abdulatif Alkhal, Muna Almaslamani, Hussam Alsoub

**Affiliations:** a Department of Infectious Diseases, Hamad Medical Corporation, P.O 3050, Doha, Qatar; b Department of Internal Medicine, Hamad Medical Corporation, P.O 3050, Doha, Qatar; c Department of Pharmacology, Hamad Medical Corporation, P.O 3050, Doha, Qatar

**Keywords:** HIV, AIDS, Opportunistic illness, Qatar

## Abstract

**Background and introduction:**

Human immune deficiency virus (HIV) infection remains a major health problem since discovery of the virus in 1981. Globally, since introduction of antiretroviral therapy, AIDS-related mortality was reduced by 47% since 2010. Also, HIV-related opportunistic infections (OIs) became less common, especially with use of prophylaxis to prevent such infections. In this study, we aim to assess the incidence of HIV infection and related OIs in Qatar for 17-year period, and to assess the spectrum of these infections, risk factors and treatment outcomes.

**Methods:**

This is a retrospective cohort study for all HIV infected patients registered in the state of Qatar from 2000 to 2016. Incidence of HIV infection and related opportunistic illness was calculated per 100,000 population. Demographic and Clinical characteristic were compared between two groups of patients with and without opportunistic illness.

**Results:**

of 167 cases with HIV infection 54 (32.3%) had opportunistic illness. The average incidence rate of HIV infection over 17 years is 0.69 per 100,000 population, and the incidence rate for opportunistic illness is 0.27 per 100,000 population. The most common opportunistic illness is pneumocystis jirovecii pneumonia (PCP) which constituted 25% of cases, followed by cytomegalovirus (CMV) retinitis 7.2%, Tuberculosis 5.4%, Toxoplasmosis 4.2% and, less than 2% for each of Kaposi sarcoma, lymphoma and cryptococcal infection.

The outcome of treatment of cases with opportunistic illness showed cure rates of 59.3%, one year relapse rates of 8.76% and overall, 90-day mortality of 3.7% however, 33.4% of patients left the country before completion of therapy.

Most of our patients in both groups were of young age, majority males, and almost half of them were Qatari. The CD4 count, CD4%, CD4/CD8 ratio and viral load were statistically significant risk factors in cases with opportunistic illness with a p value < 0.05, however presence of comorbidities was lower in patients with opportunistic illness P value of 0.032.

**Conclusion:**

Qatar has a low prevalence rate for HIV infection and related opportunistic illness. Early diagnosis and use of antiretroviral therapy are important measures to decrease the rate of opportunistic illness.

## Highlights

• HIV is a widely spread infection with special interest to public health.
• HIV-related morbidity is associated with a large burden to health systems.
• It is associated with opportunistic infections that affect all body systems.
• Population-based studies are essential for understanding its complications.

## 1 Introduction

Human immune deficiency virus (HIV) infection remains a major health problem since discovery of the virus in 1981. The World Health Organization (WHO) reported that an estimated 37.7 million people were living with HIV by the end of 2020, with an average of 680,000 people dying in 2020 from HIV-related causes [1]. However, overall, the rate of new HIV infection continues to decline in many countries [2].

Globally, since introduction of antiretroviral therapy, acquired immunodeficiency syndrome (AIDS) related death reduced by 64% since the peak in 2004 and by 47% since 2010. In 2020, 73% of all people living with HIV had access to treatment [2].

Therefore, HIV related opportunistic infections (OIs) are less common, especially with use of prophylaxis to prevent such infections [3]. Many HIV infected patients now living longer with adherence to antiretroviral medication [4].

The incidence and spectrum of OIs have been reported from many developed countries [5], but few data have been reported from middle east. In gulf region, a study from Oman showed 58% of HIV infected patients had OIs, of which pneumocystis jirovecii pneumonia (PCP) and cryptococcal meningitis were more common [6].

In Qatar, the first case of HIV was diagnosed in 1984 and antiretrovirals therapy were first used in 1997, since then most of new lines of medication have been made available. Between 2010 and 2015, the non- Qatari population constituted 87.3% of the total population [7]. However, females accounted for just 25% of the population due to a huge influx of male laborers.

In 2014, a study done by AlSoub et al., showed that in 306 cases of HIV infected patients in Qatar, the prevalence of AIDS-related OIs was 54% with majority of cases documented in non-Qatari patients [8].

We aim in this study to assess the incidence of HIV infection and related OIs in Qatar for 17-year period, and assess the spectrum of these infections, risk factors and treatment outcomes.

## 2 Material & methodology

This study was conducted as a retrospective cohort study to explore the incidence of HIV infection and related OIs per 100,000 population in Qatar.

The study was done at Hamad Medical Corporation (HMC), the main governmental health care institute in Qatar. All HIV infected patients are being followed at compromised host clinics at Communicable Disease Centre, which is a facility at HMC.

This study was registered and approved by Hamad Medical Corporation, Medical Research Centre (MRC). Registration and MRC number **16268/16**. All methods were performed in accordance with the relevant guidelines and regulations by MRC and the declaration of Helsinki. The research was retrospectively registered.

Data review of all medical records of patients diagnosed with HIV between January 2000 to December 2016.This include demographic data, clinical characteristic, viral load, CD4 count, percentage, and comparing these data between patients with and without OIS.

The spectrum of OIS, treatment outcome, use of antiretroviral therapy and prophylaxis were assessed.

Definitions:

We focused on pneumocystis jirovecii pneumonia (PCP), tuberculosis (TB), cytomegalovirus infections (CMV), cryptosporidium infection, toxoplasmosis, mycobacterium avium complex (MAC), Progressive multifocal leukoencephalopathy (PML), lymphoma &Kaposi sarcoma.

### 2.1 Statistical analysis

Descriptive statistic in terms of counts and percentages for categorical variables and mean and standard deviation or median with range (wherever appropriate) for internal variables was performed. Chi square test to see significant differences of proportions of categorical variables with opportunistic infections and student *t*-test or mann whitney *U* test for internal variable were performed. To see risk factors for opportunistic infections, multivariate logistic regression analysis was performed separately for each infection. P value 0.05 (two tailed) was considered as a statistically significant level. SPSS22.0 statistical package was used for the analysis.

## 3 Results and outcomes

Of the 339 HIV identified patients, 172 were excluded due to insufficient medical records as a result of patient's death or travelling back to their original countries. Over the seventeen years study period, analysis was performed on 167 HIV infected patients, the incidence of HIV infection and related opportunistic illness was calculated per 100,000 person-years.

**Fig. 1:**
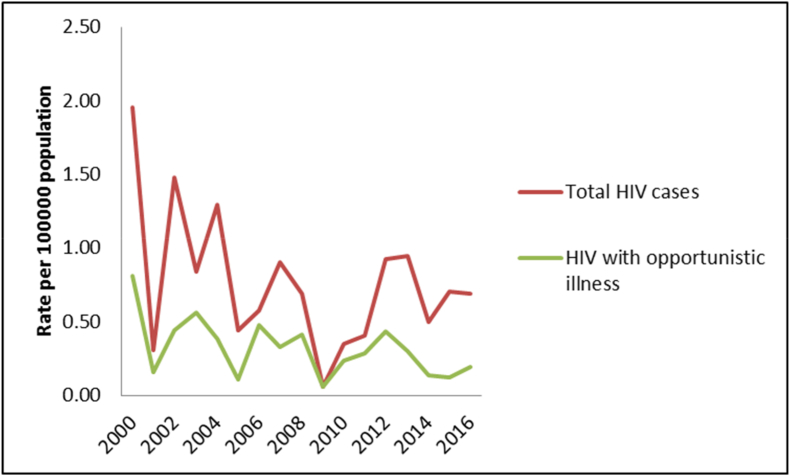
Incidence rate of HIV infection over 17 years in the state of Qatar.

The average incidence rate of HIV infection over 17 years was 0.69 per 100,000 population, whereas the incidence rate for opportunistic illness was 0.27 per 100,000 population (Fig. 1).

Demographic and Clinical characteristic were compared between two groups of patients with and without opportunistic illness. Most of our patients in both groups were young age, majority males, and almost half of them were Qatari. Patients age at the time of HIV diagnosis was lower in the group of patients without opportunistic infection (33.65 and 39.72, P value 0.002).

The CD4 count, CD4%, CD4/CD8 ratio and viral load were statistically significant risk factors in cases with opportunistic illness p value < 0.05, however presence of comorbidities was lower in patients with opportunistic illness P value0.032 (Table 1).

**Table 1:** Demographic data, CD4%, absolute CD4 count, and CD4/CD8 ratio between HIV-infected patients with and without opportunistic illness.

	HIV with opportunistic illness (n = 67)		HIV without opportunistic illness (n = 100)		P value
	Mean	Standard Deviation	Mean	Standard Deviation	
**Age at diagnosis**	39.72	11.246	33.65	12.920	0.002^a^
**CD4**	99.00	121.542	574.36	427.218	<0.001^a^
**CD4 percentage**	5.48	6.026	20.80	12.844	<0.001^a^
**CD ratio**	0.13	0.137	0.76	1.731	0.023^a^
	Frequency	%	Frequency	%	P value
**Nationality (n=167)**					
Qatari	32	47.8%	48	48.0%	0.976
Non-Qatari	35	52.2%	52	52.0%	
**Gender (n=167)**					
Male	51	76.1%	75	75.0%	0.869
Female	16	23.9%	25	25.0%	
**Marital status (n=167)**					
Single	22	32.8%	42	42.0%	0.349^a^
Married	43	64.2%	57	57.0%	
Divorced	2	3.0%	1	1.0%	
**Mode of transmission (n=167)**					
Sexual transmission	57	85.07%	90	90.0%	0.336
Others	10	14.93%	10	10.0%	
**Hepatitis C infection (n=167)**	2	3.0%	3	3.0%	0.853
**Hepatitis B infection (n=167)**	3	4.5%	2	2.0%	0.610
**Co-morbidities (n=167)**	15	22.4%	24	24.0%	0.032^a^
**Viral load (n=167)**					
<100000	25	25.8%	72	74.2%	<0.001^a^
>100000	36	64.3%	20	35.7%	

a Indicate statistically significant result.

**Fig. 2:**
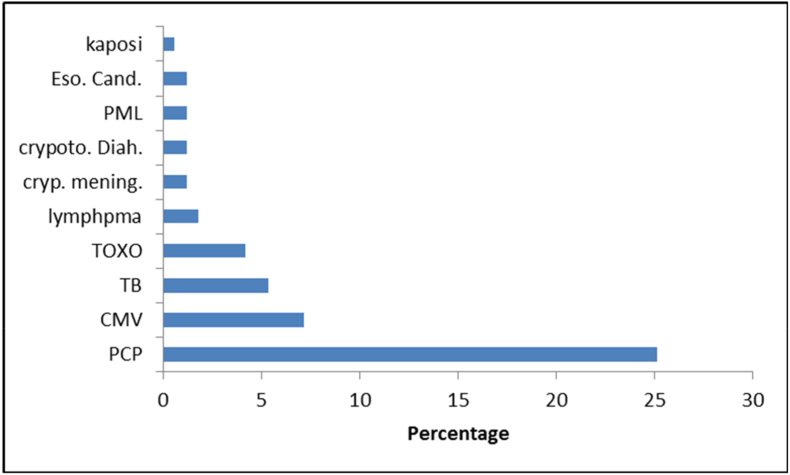
Type (Percentage) of opportunistic illnessness among HIV cases. **Acronym: Eso. Cand.:** Esophageal candidiasis. **PML:** Progressive mulitifocal leukoencephalopathy **crypoto.Diah.:** Cryptosporidiosis diarrhea. **cryp. mening.:** Cryptococcal meningitis. **TOXO. Cns.:** Toxoplasmosis. **TB:** Tuberculosis. **CMV:** Cytomegelovirus infection**. PCP:** Pneumocystis jirovecii pneumonia.

The most common opportunistic illness is pneumocystis jirovecii pneumonia (PCP) 25% (42 of cases). Followed by CMV retinitis 7.2%, tuberculosis 5.4%, toxoplasmosis 4.2% and, less than 2% for each of Kaposi sarcoma, lymphoma and cryptococcal infection (Fig. 2).

Assessment of the outcome of management of the opportunistic illness showed cure rates of 59.3%, one year relapse rates of 8.76% and overall, 90-day mortality of 3.7% however, 33.4% of patients left the country before completion of therapy.

## 4 Discussion

Since the initial epidemic of HIV, the overall incidence of new HIV case was declining globally. New HIV infections have been reduced by 52% since the peak in 1997, with 36.3 million people dying from AIDS-related illnesses since the start of the epidemic [2].

Qatar is a small country in the gulf area with an expanding population, growing fast over the last 10 years, current population in 2020 reached around 2.8 million, 25% of which were Qataris. As per Ministry of Development Planning and Statistic, total population in the State of Qatar amounted to 2,404,776 in 2015; a rise of 41.5% compared to 2010, with an annual increase of 6.9%. Males and females accounted for 75.6% and 24.4% respectively [9].

We notice from our study the declining number of newly diagnosed HIV infected cases despite the rise in population, which can be explained with the overall decline of HIV incidence [2].

Also, the incidence of HIV related OIs is declining, explained by the use of antiretrovirals medication and prophylaxis [3].

In Qatar, the use of HIV screening programs for all new immigrants, premarital individuals, antenatal screening, and pre-employment screening for selected jobs, have contributed to the early diagnosis of HIV infections and the reduced rate of OIs. In addition, HIV screening test is done for all expatriates who will stay more than one month in the country.

HIV treatment is available for all of our patients, which also contributed in lowering the incidence of opportunistic infections among them.

In our study, Patients with OIs have significant decrease in CD4 count and CD4/CD8 ratio with high HIV viral load compared to patients without infections, with the same observation being documented in other studies [[11], [12], [13]].

We also noticed a smaller number of hepatitis B or C combined infections among HIV cases, which could be explained by blood donation screening program, along with low incidence of drug abusers among our HIV infected patients.

We observed the presence of comorbidities among our HIV cases, mainly diabetes, which may be related to overall high incidence of diabetes among Qatari population [10].

PCP remains the most common type of OIs, accounting for 25% (42 cases) of all diagnosed OIs in our study. Studies from other regional countries show similar prevalence; Oman 25% and Bahrain 15.1% [6,11]. A definitive diagnosis of PCP with demonstration of the organism in induced sputum samples or broncho-alveolar lavage (BAL) fluid was made in most of our studied cases. A total of 16 patients (9.6%) with HIV/AIDS had PCP as an AIDS-defining OI at their first presentation.

CMV disease/retinitis was the 2nd most common OI in our study representing 7.2% (12 cases of infected HIV/AIDS patients). Diagnosis of the disease was made based on positive CMV serology (IgM,IgG), high PCR titer and fundoscopic findings. In Oman, however, 17% of the HIV infected patients were diagnosed with CMV retinitis [6].

Although tuberculosis is a common disease in the region due to high number of workers from highly endemic areas, combined HIV and TB cases were low 5.4% (9 cases). This could be explained by the fact that all new immigrants to Qatar undergo routine HIV screening and TB screening (Chest-X-ray and serum QuantiFERON test). Another explanation is the early diagnosis of TB, initiation of TB treatment immediately upon confirmation of the diagnosis, and treatment of latent TB infection. We observed that none of the studied cases had MDR TB (multi-drug resistant Tuberculosis). This can be partially explained by the low overall prevalence of MDR TB in Qatar [14].

Toxoplasmosis, caused by Toxoplasma *gondii*, is one of the major OIs afflicting HIV patients. Cerebral toxoplasmosis is the most common cause of focal neurological disorders in HIV patients. In our cohort, cerebral toxoplasmosis was found in 4.2% (7 out of 167) of HIV infected patients. In a study from Oman, cerebral toxoplasmosis was reported in 12% of the HIV-infected patients [6].

Cryptococcus neoformans is one of the causes of invasive fungal disease in patients with HIV worldwide. Meningitis is the commonest clinical manifestation of invasive cryptococcosis infection. In our study, Cryptococcus meningitis accounted for only 1.2% of all HIV patients. As a result, primary prophylaxis for invasive cryptococcal disease is not a common practice in Qatar.

Similar low incidence was observed in Indian reports (6–8%), whereas it is about 5–11% in the USA, 33% in Africa, and 28.5% in Thailand [15]. Interestingly, in Oman, reports estimate the incidence of cryptococcal meningitis as high as 21% of all HIV infected patients. The exact explanation for such high incidence in Oman is unclear [6].

Cryptosporidium infection was observed in only 2% in our study. Regional data showed similar low incidence, for example, in Oman it was 3% [6]. This is in contrast to data from Ethiopia where 21% of HIV patients had Cryptosporidium parvum infection [16]. In patients with HIV, cryptosporidiosis usually causes chronic diarrhea, however, it may cause potentially fatal complications, like bile duct perforation [17]. Of interest, the rate of cryptosporidiosis has subsided in many countries because of the use of ART [18].

Kaposi sarcoma and Progressive Multifocal Leukoencephalopathy (PML) were rare opportunistic infections in our study group and worldwide. According to one study, we have a smaller number of patients with cryptococcal meningitis in comparison to Oman [6]. In our study none of the patients had Mycobacterium Avium Complex (MAC) infection out of all 167 HIV-infected patients. This can be explained by routine use of primary prophylaxis in patients with CD4 count less than 50.

The percentage of treatment success among patients with OIs in our study is 59.3%, however, one third of patients lost follow up as they left the country.

## Conclusion

Our study from Qatar of nearly 167 cases of HIV-infected patients from all age groups, over a period of 17 years, demonstrated lower incidence rate of HIV infection and related opportunistic illness compared to other parts of the world. The implementation of HIV screening programs has led to an early diagnosis of HIV infection and early use of antiretroviral therapy and prophylaxis, which are important measures to decrease the rate of opportunistic illness and HIV-related morbidity and mortality.

We hope that our study will serve as a date base and as a bridge for future, and wider studies related to epidemiologic research in the region on HIV and AIDS-defining opportunistic infections.

## Strength of the study

This is the largest retrospective cohort study on HIV and AIDS-defining opportunistic infections performed up to date in the state of Qatar. The study includes patients from a widely diverse population including patients from all over the world.

## Limitations of the study

33.4% of patients left the country before completion of therapy, therefore long-term follow up was not established in these patients.

This manuscript has been formatted in line with the STROCSS Guidelines [19].

## Ethical approval

Approval from the Hamad Medical Corporation Medical Research Council was obtained prior to submission of this manuscript. Study reference 16268/16.

## Sources of funding

This article did not receive any specific grant from funding agencies in the public, commercial, or not-for-profit sectors.

Open access funding will be provided by Qatar National Library (QNL).

## Author contribution

Maisa Ali (MA) wrote the manuscript and initial research protocol. Almurtada Razok (AR) performed critical review and edited the manuscript. Mahmoud Gasim (MG) performed data analysis and designed the tables and figures. Nada Elmaki (NE) performed data collection. Wael Goravy (WG) performed data collection. Abdulatif Alkhal (AA) wrote the inclusion and exclusion criteria and provided epidemiologic data. Muna Almaslamani (M.A) revised the manuscript. Hussam Alsoub (H.A) was the mentor of the research, supervised the process and participated in manuscript writing. All authors contributed and approved the final version for submission.

## Registration of research studies

This research was registered in and approved by Hamad Medical Corporation Medical Research Council (MRC) with unique number 16268/16 and in accordance with the Declaration of Helsinki.

## Guarantor

Dr. Almurtada Razok.

## Declarations

Research registration, Ethical approval, and consent to participate: An approval from Hamad Medical Corporation, Medical Research Centre (MRC) was obtained prior to submission of this manuscript. **MRC number 16268/16**. All methods were performed in accordance with the relevant guidelines and regulations by MRC and the declaration of Helsinki.

## Consent for publication

Written informed consents were obtained from the patients for publication of this cohort study and the accompanying information. Copies of the written consents are available for review by the Editor-in-Chief of this journal on request.

## Availability of data and materials

The datasets used and/or analysed during the current study are available from the corresponding author on reasonable request.

## Provenance and peer review

Not commissioned, externally peer-reviewed.

## Declaration of competing interest

None to be declared.
